# Supplementary material for: Seminal Plasma Antioxidants Are Related to Sperm Cryotolerance in the Horse
Source: Antioxidants (Basel). 2022 Jun 28;11(7):1279. doi: 10.3390/antiox11071279 (PMC9311553; doi:10.3390/antiox11071279)
Supplement: Supplementary file 1 [file antioxidants-11-01279-s001.zip › antioxidants-1776649-supplementary.pdf]

Table S1. Mean ± SEM and range of sperm motility parameters in horse ejaculates classified as of good (GFE, *n* = 13) or poor freezability (PFE, *n* = 8) recorded post-thaw.

Parameter	GFE		PFE	
	Mean ± SEM	Range	Mean ± SEM	Range
TM (%)	66.44 ± 2.61	55.38–85.84	35.54 ± 4.36	8.60–48.54
PM (%)	32.65 ± 2.01	23.05–48.06	16.28 ± 3.34	1.67–29.01
VCL (µm/s)	163.11 ± 3.41	138.70–180.53	128.75 ± 7.93	85.94–160.43
VSL (µm/s)	55.31 ± 1.45	45.77–63.28	42.59 ± 4.32	20.93–56.76
VAP (µm/s)	75.86 ± 1.64	65.51–84.85	59.89 ± 3.94	36.61–72.25
LIN (%)	32.13 ± 0.85	26.69–37.81	30.61 ± 1.76	23.14–36.37
STR (%)	67.60 ± 1.26	61.55–74.47	64.66 ± 3.41	51.08–74.81
WOB (%)	46.45 ± 0.81	40.83–49.63	46.84 ± 0.82	43.56–49.38
ALH (µm)	2.19 ± 0.05	1.92–2.49	1.80 ± 0.10	1.22–2.22
BCF (Hz)	26.66 ± 0.88	22.41–33.16	23.22 ± 2.09	14.44–30.14

TM (%): total motility; PM (%): progressive motility; VCL (µm/s): curvilinear velocity; VSL (µm/s): straight line velocity; VAP (µm/s): average path velocity; LIN (%): linearity coefficient; STR (%): straightness coefficient; WOB (%): wobble coefficient; ALH (µm): amplitude of lateral head displacement; BCF (Hz): beat-cross frequency.

Table S2. Mean ± SEM and range of sperm functionality parameters in horse ejaculates classified as of good (GFE, *n* = 13) or poor freezability (PFE, *n* = 8) recorded post-thaw.

Parameter	GFE		PFE	
	Mean ± SEM	Range	Mean ± SEM	Range
SYBR14$^+$/PI$^-$ (%)	67.90 ± 1.54	60.29–76.99	42.03 ± 2.49	29.80–50.23
PNA-FITC$^-$/PI$^-$ (%)	34.08 ± 2.38	20.74–48.44	32.70 ± 4.01	7.54–44.79
JC-1$_{agg}$ (High-MMP, %)	17.85 ± 1.88	8.06–33.30	16.89 ± 1.84	6.95–24.15
DCF$^+$/PI$^-$ (%)	0.61 ± 0.11	0.10–1.36	0.84 ± 0.19	0.07–1.63
E$^+$/YO-PRO-1$^-$ (%)	9.34 ± 1.14	3.64–16.35	13.62 ± 2.78	1.92–30.08
M540$^+$/YO-PRO-1$^-$ (%)	0.56 ± 0.09	0.10–1.51	0.85 ± 0.26	0.45–2.64

SYBR14$^+$/Propidium iodide (PI)$^-$ (%): sperm with intact plasma membrane (viable sperm); *Arachis hypogaea* (peanut) agglutinin-fluorescein isothiocyanate (PNA-FITC)$^-$/PI$^-$ (%): sperm with intact acrosome membrane; 5,5′,6,6′-tetrachloro-1,1′3,3′tetraethyl-benzimidazolylcarbocyanine iodide aggregates (JC-1$_{agg}$ (High-MMP, %)): sperm with high mitochondrial membrane potential; dichlorofluorescein (DCF)$^+$/PI$^-$ (%): viable sperm with high intracellular ROS levels; ethidium (E)$^+$/1-(4-[3-methyl-2,3-dihydro-(benzo-1,3-oxazole)-2-methylidene]-quinolinium)-3trimethylammonium propane diodide (YO-PRO-1)$^-$ (%): viable sperm with high superoxides (O_2^-) levels; merocyanine 540 (M540)$^+$/YO-PRO-1$^-$ (%): viable sperm with high membrane lipid disorder.

Table S3. Mean ± SEM and range of activity levels of enzymatic and non-enzymatic antioxidants, as well as total oxidative status (TOS) and oxidative stress index (OSI) measured in seminal plasma (SP) of horse ejaculates (*n* = 21).

Group	Antioxidant	Mean ± SEM	Range
Enzymatic antioxidants	PON1 (IU/L)	0.71 ± 0.06	0.33–1.33
	SOD (IU/mL)	217.10 ± 24.72	87.00–617.00
	CAT (IU/mL)	0.22 ± 0.03	0.03–0.47
	GPX (IU/L)	66.21 ± 8.70	14.10–187.00
Non-enzymatic antioxidants	CUPRAC (mmol/L)	0.31 ± 0.02	0.15–0.51
	FRAP (mmol/L)	0.46 ± 0.04	0.20–0.88
	TEAC (mmol/L)	0.69 ± 0.06	0.27–1.39
TOS (µmol/L)		5.20 ± 0.17	4.10–6.80
OSI (arbitrary units)		8.90 ± 1.02	3.25–19.48

PON1 (IU/L): paraoxonase type 1; SOD (IU/mL): superoxide dismutase; CAT (IU/mL): catalase; GPX (IU/L): glutathione peroxidase; CUPRAC (mmol/L): cupric-reducing antioxidant capacity; FRAP (mmol/L): ferric-reducing ability of plasma; TEAC (mmol/L): Trolox equivalent antioxidant capacity; TOS (µmol/L): total oxidative status; OSI (arbitrary units): oxidative stress index.
